# Accuracy of novel custom 3D-printed metal and polymer cutting guides for segmental mandibulectomy in the dog: a cadaveric study

**DOI:** 10.3389/fvets.2026.1780938

**Published:** 2026-03-04

**Authors:** Rachel McKay, Marine Traverson, Caroline Alting, Satyanarayana Konala, Erin Perry, Angelica Luzzi, Ken Gall

**Affiliations:** 1 Department of Clinical Sciences, College of Veterinary Medicine, North Carolina State University, Raleigh, NC, United States; 2 Department of Mechanical Engineering and Materials Science, Duke University, Durham, NC, United States; 3 Center for Additive Manufacturing and Logistics, Fitts Department of Industrial and Systems Engineering, North Carolina State University, Raleigh, NC, United States; 4 Restor3d, Durham, NC, United States

**Keywords:** 3D-printing, accuracy, mandibulectomy, materials, surgical guide

## Abstract

**Introduction:**

The objectives of this study were to design a 3D-printed custom guide for segmental mandibulectomy in the dog and evaluate the impact of different materials (metal versus polymer) on the performances of the guided procedure in cadaveric dogs.

**Methods:**

Twenty canine cadaveric heads were randomized in two study groups and received bilateral segmental mandibulectomies performed with a metal or polymer surgical guide. Pre-operative computed tomography (CT) images of the skull were used to design custom 3D-printed surgical guides and were repeated after placement and osteotomy. Mean absolute linear deviation between planned and performed cuts, procedure duration, and qualitative assessment were compared.

**Results:**

Polymer guides were associated with easier (*p* =0.020) and faster (*p* =0.004) placement. No incidence of failure was recorded when using metal guides, whereas 30 and 15% of polymer guides experienced cracking and fissuring, respectively (*p* =0.001). Dorsal displacement and gap formation between guide and mandibular body was noted in 7/20 metal guides on CT. Mean absolute linear deviation between planned and performed cuts was not significantly different between material groups (*p* =0.612). Polymer guides presented several advantages including efficient placement despite a high incidence of material failure. Difficulty of placement encountered with metal guides relates to the rigidity of the material. However, these limitations did not have any significant impact on surgical accuracy.

**Discussion:**

Overall, the study did not demonstrate any difference in accuracy between materials but highlighted differences in performance specific to each material. Thus, surgical guide manufacturing and material choice could be tailored to specific clinical applications, whether strength/durability or flexibility/conformability is favored.

## Introduction

Additive manufacturing (AM), commonly known as 3D-printing, is gaining popularity in both human and veterinary surgery, with applications ranging from the production of patient specific anatomic models and implants to the design of custom surgical guides (1, 2). Using AM, three-dimensional objects are built layer by layer from digital models derived from patient-specific diagnostic images. Among its clinical applications, one area of particular interest and ongoing research is the development of custom surgical guides to improve surgical precision and outcomes. Patient-specific 3D-printed surgical guides have been shown to enhance accuracy in a variety of procedures, including orthopedic, neurologic, and oromaxillofacial surgeries in both human and veterinary fields (2–10). These guides provide specific advantages such as enabling precise measurements in deformity correction (4) or fracture repair (11), preserving critical anatomic landmarks (9, 12), and defining surgical margins in oncologic resections (2, 3, 13–15).

A previous study evaluating custom maxillectomy guides in the dog demonstrated improved procedural accuracy, with a highest mean linear deviation of <2 mm when using guides compared to >5 mm with freehand technique (3). Notably, the guide enabled a novice surgeon to achieve accuracy comparable to that of an experienced surgeon, despite the complexity of the procedure. Multiple factors are thought to influence the accuracy of surgical guides, including surgeon experience, computer-aided design and manufacturing, anatomic location, guide support, procedural complexity, and the interval between design and surgery (2, 16). The total error or deviation from the intended cut plane when using a 3D-printed cutting guide may arise from any stage of the process - design and manufacturing, intraoperative positioning, or the cutting itself. The accumulation of these errors can result in displacement and/or angular deviations, ultimately reducing surgical precision, altering surgical margins, or causing implant mismatch. In the cited study, a trend toward greater error in guided cutting compared to guided positioning was observed when a polymer guide was used for maxillectomy in the dog (3), potentially reflecting micromotion due to guide design, anatomic support, or material flexibility. Thus, one area warranting further investigation for optimizing patient-specific guide design and accuracy is the selection and performance of 3D-printed materials.

Polymer materials, such as acrylate-based resins or powder-based nylon materials, are most commonly used for surgical guide manufacturing, while only a few studies have reported the use of metal for this purpose (17–19). To the authors’ knowledge, no studies have directly assessed the effect of material composition (metal versus polymer) on surgical guide accuracy. The objectives of this study were to (1) design a 3D-printed custom guide for segmental mandibulectomy in the dog, and (2) compare the performance of metal versus polymer materials in guided procedures in dogs. We hypothesized that metal surgical guides would achieve greater accuracy than polymer guides.

## Materials and methods

### Specimen randomization and study groups

Twenty cadaveric heads were obtained from dogs euthanized at local shelters for reasons unrelated to this study. Specimens were thawed for 18–24 h prior to each procedure. Each head was classified as dolicho-, brachy-, or mesocephalic, and head length was measured from the maxillary canine tooth to the caudal aspect of the occipital bone.

For experimental purposes, left and right mandibles were treated as independent subjects, yielding twenty mandibles per treatment group. Heads were numbered, lateralized, and randomized into two treatment groups: segmental mandibulectomy procedures performed with either (1) metal 3D-printed surgical guides or (2) polymer 3D-printed surgical guides.

Procedures were carried out by one of two surgeons: a board-certified surgical oncologist (MT; experienced surgeon) or a second-year surgical resident (RM; novice surgeon). Within each treatment group, specimens were further randomized according to surgeon experience. Of the 20 metal-guided procedures, 10 were performed by the experienced surgeon and 10 by the novice surgeon; the same distribution was applied to the polymer-guided procedures. Each surgeon assisted during the procedures performed by the other.

### Mandibulectomy planning

**Figure 1:**
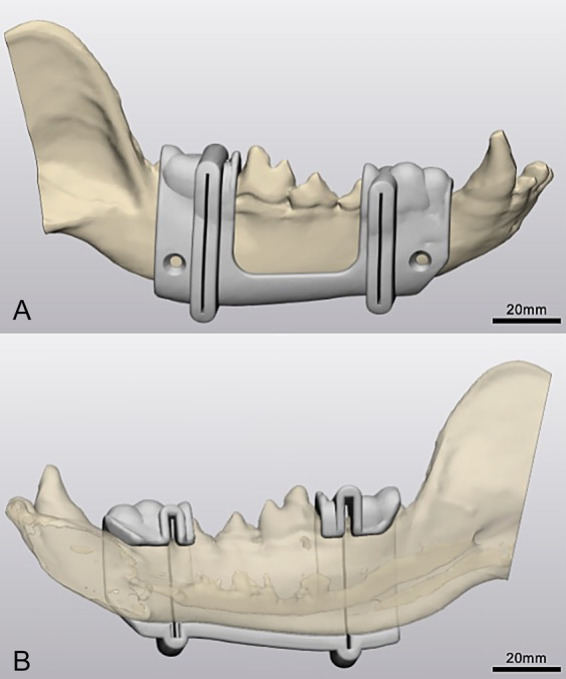
Three-dimensional design. **(A)** Three-dimensional model of a cadaveric mandible in a dog with computer-aided design (CAD) of a custom mandibulectomy guide in Materialise 3-matic (lateral view). **(B)** Medial view of 3D-printed surgical guide highlighting mandibular canal and osteotomy planes in Materialise 3-matic.

Pre-operative computed tomography (CT) imaging was performed for all cadaver heads using a 64-slice CT scanner (Siemens 64 slice; Siemens Medical Solutions, Malvern, Pennsylvania). The resulting images were imported into DICOM-compatible image processing software (Mimics Innovation Suite; Materialise, Leuven, Belgium). Two osteotomy planes were defined to complete the segmental mandibulectomy: the first immediately distal to the second premolar tooth (P2) and the second immediately distal to the first molar tooth (M1). The osteotomy locations were planned preoperatively using CT imaging, with specific attention to avoiding teeth and ensuring precise placement between adjacent tooth roots. Each skull was thresholded, segmented, and virtually planned using Mimics and 3-Matics softwares (Mimics Innovation Suite; Materialise, Leuven, Belgium) (Figure 1).

### Custom guides design and manufacturing

Custom 3D-printed surgical guides were designed in Materialise 3-Matics software in collaboration with a PhD candidate in engineering (CA). Each guide was tailored to fit the contours of the mandible and dentition of the individual specimen. The surgeon (MT) and surgery resident (RM) defined the locations of the two virtual osteotomy planes as well as screw hole placement (positioned just ventral to the mandibular canal) based on surgical anatomy. Guides were then designed by two veterinary students (EP, AL) with assistance from the PhD candidate (CA). The placement of datum planes and screw-hole cylinders was reviewed by the surgical team prior to further development.

Cutting slots (0.7 mm in width) were incorporated to provide appropriate tolerance, and guides were designed with sufficient material for stability while respecting anatomic constraints of the jaws and surrounding soft tissues, as well as leaving central space to simulate tumor mass effect. For initial positioning, the surrounding teeth (P1, P2, M2 +/− M3) served as anchoring landmarks (Figure 1).

**Figure 2:**
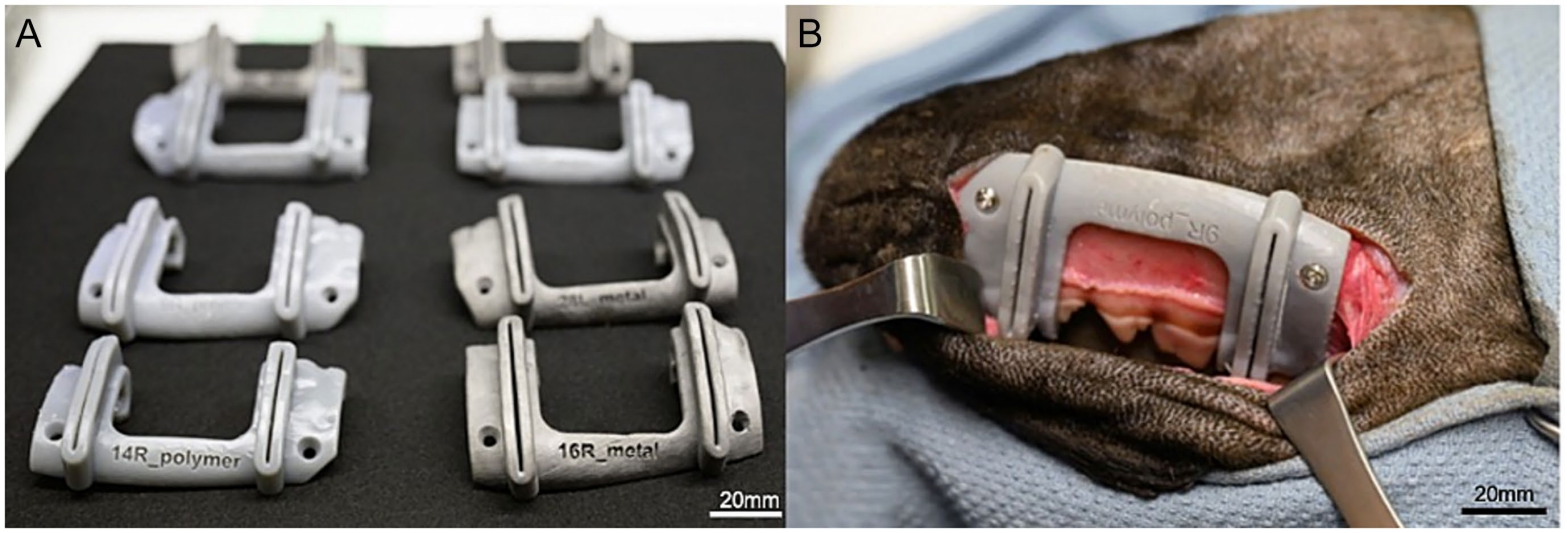
Three-dimensional manufacturing **(A)** 3D-printed surgical guides for segmental mandibulectomy manufactured in polymer augmented with barium (left) and titanium (right). **(B)** Polymer guide secured to a cadaveric mandible (ventrolateral view) of a dog. The guide conforms to the contours of the skull, dental quadrant, and gingiva, and incorporates two drilling holes for cortical screw placement ventral to the mandibular canal to provide stabilization, along with two cutting grooves to guide the rostral and caudal osteotomies between P2–P3 and M1–M2, respectively.

Metal cutting guides were manufactured via laser-powder bed fusion (3D Systems DMP ProX 320 printer, Rock Hill, South Carolina) using titanium alloy (grade 23 ELI Ti6Al4V). Polymer guides were fabricated using a Form4 stereolithography (SLA) printer (Formlabs, Boston, Massachusetts) with Tough 1,500 resin (Formlabs, Boston, Massachusetts) doped with 5% barium sulfate, selected for its biocompatibility, dimensional accuracy, radiopacity, and compatibility with steam autoclaving, sterilization by irradiation, and ethylene oxide sterilization (Figure 2).

### Surgical procedures

All segmental mandibulectomy procedures were performed via a ventral approach as previously described by de Mello Souza et al. (20). A skin incision was made beginning at the caudal aspect of the mandibular symphysis and extended caudally to the level of the angular process of the mandible. The platysma and mylohyoideus muscles were transected to expose the mandibular body, and the remaining soft tissues were dissected from the mandible. The oral mucosa was then incised to complete exposure.

Each surgical guide was positioned on the mandible and secured with two 2.7-mm stainless steel cortical screws (Figure 2). Osteotomies were performed using a Hall oscillating saw (Conmed, Largo, Florida) with a 0.6-mm kerf blade for both cuts. Following completion of both cuts, the screws and guide were removed, and the segmental mandibulectomy specimen was extracted.

**Figure 3:**
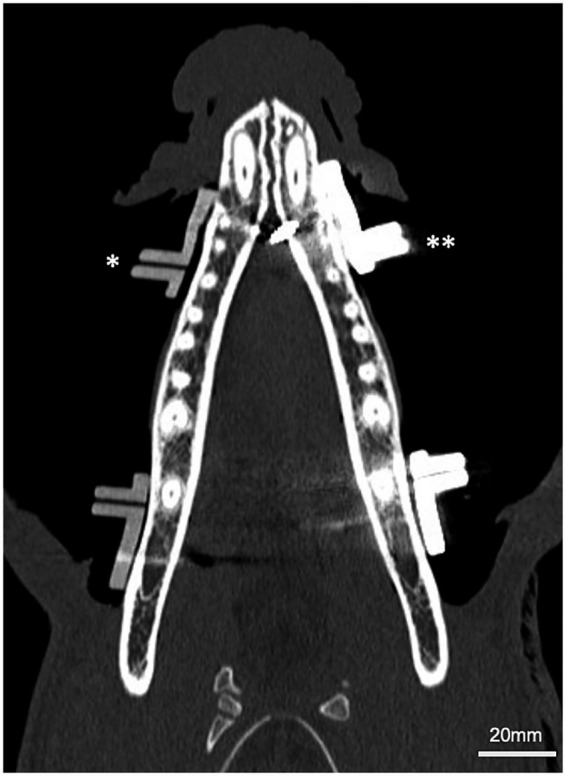
Tomodensitometric evaluation after guide placement. Dorsal CT view of mandibles of dogs following guide placement, demonstrating polymer (single asterisks, *) and titanium (double asterisks, **) guides secured to mandibular body.

Computed tomography (CT) scans were repeated after guide placement and again following osteotomy for deviation analysis (Figure 3). Measurements were obtained after each scan at the point of greatest gap between the medial surface of the guide wall and the lateral surface of the mandibular body to assess guide fit. Additionally, the mean attenuation of each guide was recorded in Hounsfield units (HU), and the number of screw cortices engaged was documented.

### Qualitative assessment

The ease of guide placement was recorded for each procedure and categorized as easy (guide fitted within 30 s without modification of the surgical approach), moderate (guide placement required modification of the surgical approach and/or fitting time between 30 s and 2 min), or difficult (guide could not be fitted or stabilized without approach modification and/or required >2 min to fit).

For all groups, the quality of the osteotomy was graded as high (both cuts smooth, straight and complete), moderate (one cut irregular or incomplete), or low (both cuts irregular or incomplete).

Debris in surrounding soft tissues post-osteotomy was subjectively assessed as mild (debris limited to osteotomy edges), moderate (debris along osteotomy edges with extension into soft tissue), or severe (debris covering all adjacent soft tissues). Soft tissue trauma was also classified as mild (injury to the gingiva, musculature, tongue, lip, or salivary gland requiring no intervention), moderate (trauma necessitating sutures to repair but without functional impairment), or severe (extension trauma to gingiva and musculature, with potential salivary duct transection and possible functional impairment).

Finally, guide failure was recorded in cases of inability to fit the guide, cracking of the saw groove, cutting through the groove, unstable or loose placement, breakage of the guide prior to completion of the osteotomy, or inability to secure the guide with screws.

### Efficiency

For all groups, the duration of each procedural step was recorded, excluding the time required for the surgical approach. Total mandibulectomy time was defined as the interval from guide placement through osteotomy completion to bone segment removal. Guide preparation time was defined as the sum of the time required to place the guide and to secure it with screws. Similarly, the time required to perform the guided cuts and the time to remove both the guide and bone segment were recorded separately and included in the total osteotomy time.

### Accuracy and error analysis

Accuracy between groups was assessed using mean absolute linear deviation between planned (pre-operative), guided (post-guide placement), and performed (post-mandibulectomy) osteotomies. CT scans were obtained pre-operatively to plan the osteotomies, and repeated following guide placement and after osteotomy to evaluate sources of deviation. All CT datasets were uploaded and aligned in Geomagics (Artec 3D, Senningerberg, Luxemborg). Prominent anatomic landmarks (e.g., mandibular condyle, dentition) were manually selected using local multipoint alignment to achieve model registration. In 3-Matic, osteotomy planes were generated via the three-point method, and planes and surfaces were manually marked and exported as STL files.

**Figure 4:**
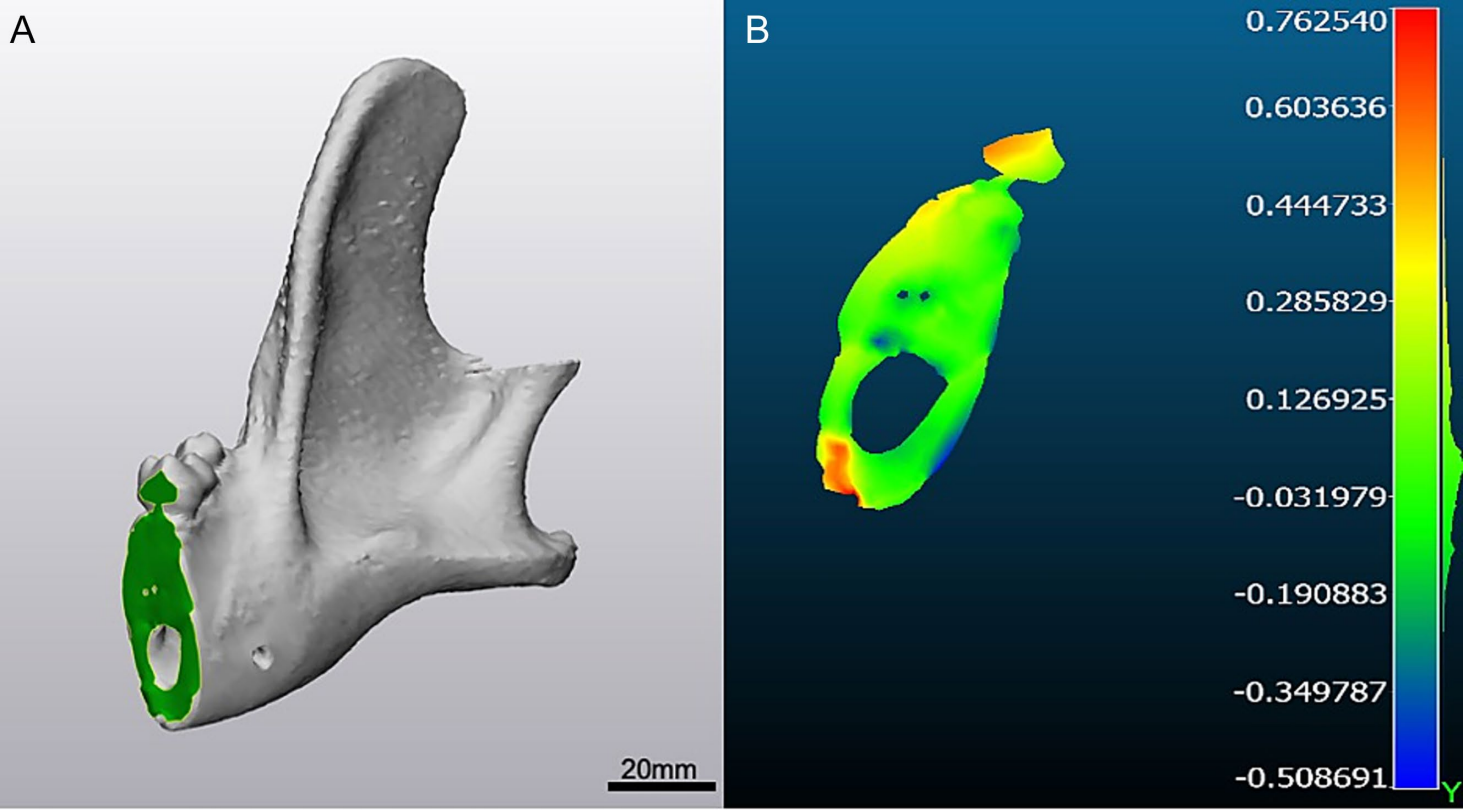
Deviation analysis. **(A)** Cut surface of caudal mandibulectomy osteotomy depicted in 3-matic. **(B)** Heatmap generated in Cloud Compare highlighting the cutting error observed along the caudal cut from planned to performed osteotomy. In this case, the cutting error was negative, or towards the defect, with error pictured in colors. Color bar units are in millimeters.

The STL surfaces of each cut were then converted into point clouds and analyzed in CloudCompare (open-source software) as previously described (3, 21). A best-fit plane was generated for each surface, and the distance of each point in the cloud to its corresponding plane was calculated using the “distance to primitive” function. Histograms and heatmaps of these distances were generated for subsequent analysis (Figure 4).

To determine the source of deviation, three error values were recorded. *Total error* was defined as the deviation between the planned and performed cuts. *Positioning error* was defined as the deviation between the planned and guided cuts, reflecting inaccuracy from CAD/CAM and/or guide placement. *Cutting error* was defined as the deviation between the guided and performed cuts, reflecting micromotion or guide failure during osteotomy. *Positioning error* was calculated by subtracting *cutting error* from *total error*.

### Statistics

Each subgroup included 10 mandibulectomy procedures, based on a prior power analysis (3) that established a minimum sample size of 8 specimens per treatment group for *α* = 0.05 and *β* = 0.80. Previous human literature on the accuracy of 3D-printed surgical guides in maxillofacial and dental surgery was also referenced, reporting an expected mean linear deviation of 2 mm (3, 22–24).

For all recorded times (total mandibulectomy, guide placement, guide securing, cutting, and removal), mean and standard deviation values were calculated. Linear deviations were reported as mean absolute values with standard deviations. Statistical comparisons between groups were performed using a Student’s *t*-test or, when the normality assumptions were not met, Wilcoxon rank-sum test.

## Results

### Specimens

The mean cadaver head length, measured from the maxillary canine tooth to the caudal aspect of the occipital bone, was 22.8 cm (range,18–28 cm). Two specimens were classified as dolichocephalic, while the remaining 38 were mesocephalic. No significant differences in head length were observed between material groups (*p* = 0.133) or between surgeon group (*p* = 0.376).

### Design and manufacturing

Marked differences in manufacturing time were observed between materials, with polymer guides requiring substantially less time than metal guides across printer preparation, build, and post-processing stages. Preparation of the SLA printer (Form4, Formlabs Inc) required approximately 5 min, including cleaning the build platform, inserting the resin cartridge, and preparing the resin tank. In contrast, setup of the metal printer (ProX DMP 320, 3D Systems Inc) required approximately 5 h, which included cleaning the printer interior with IPA, sieving the Ti6Al4V powder, preparing the wiper blade, inserting the build plate, purging the chamber with argon, and completing the necessary print-readiness cycles.

Differences were also noted in the machine time required for each printing method. A single polymer build plate accommodated four surgical guides and required 2 h of machine time; therefore, producing 20 polymer guides required 5 build plates and a total of 10 h. In contrast, one metal build plate accommodated 10 guides and required 20 h of machine time, with two build plates needed to produce 20 metal guides and resulting in a total machine time of approximately 40 h.

Post-processing time also differed between the two printing methods. For the polymer guides, post-processing consisted of 30-min IPA followed by 1 h of UV curing. After curing, minor filing was performed to smooth the touchpoints left by the printing supports, requiring an additional 30 min. In total, each polymer build plate required 2 h of post-processing. In contrast, post-processing for the metal guides was considerably more time intensive. Electronic discharge machining (EDM) was used to remove the parts from the build plate and required approximately 5 h per plate. Support removal and deburring of touchpoints using a Dremel required an additional 6 h, followed by aluminum oxide blasting, to achieve final surface finish, which took 2 h. Altogether, one build plate of metal surgical guides required approximately 13 h of post-processing.

In total, one build plate of polymer guides required 4 h of manufacturing, whereas one build plate of metal guides required 28 h. Producing 20 polymer guides required four build plates, resulting in a total manufacturing time of 16 h. In contrast, producing 20 metal guides required 2 build plates, yielding a total manufacturing time of 56 h.

### Surgical procedures

All segmental mandibulectomies were performed as planned via a standard ventral approach to the mandible, followed by timed placement of custom guides. Guides were secured using 2.7 mm cortical screws of varying lengths, selected based on implant availability. Each procedure involved two osteotomies performed through the guide cut slots. When using the metal guide, the narrow slot geometry and rigidity of the material created friction between the saw blade and the guide walls, leading to rapid blade dulling and need for frequent replacement. The saw battery likewise required recharging after only 3–4 osteotomies, which is considerably fewer than typically expected in a clinical setting.

**Figure 5:**
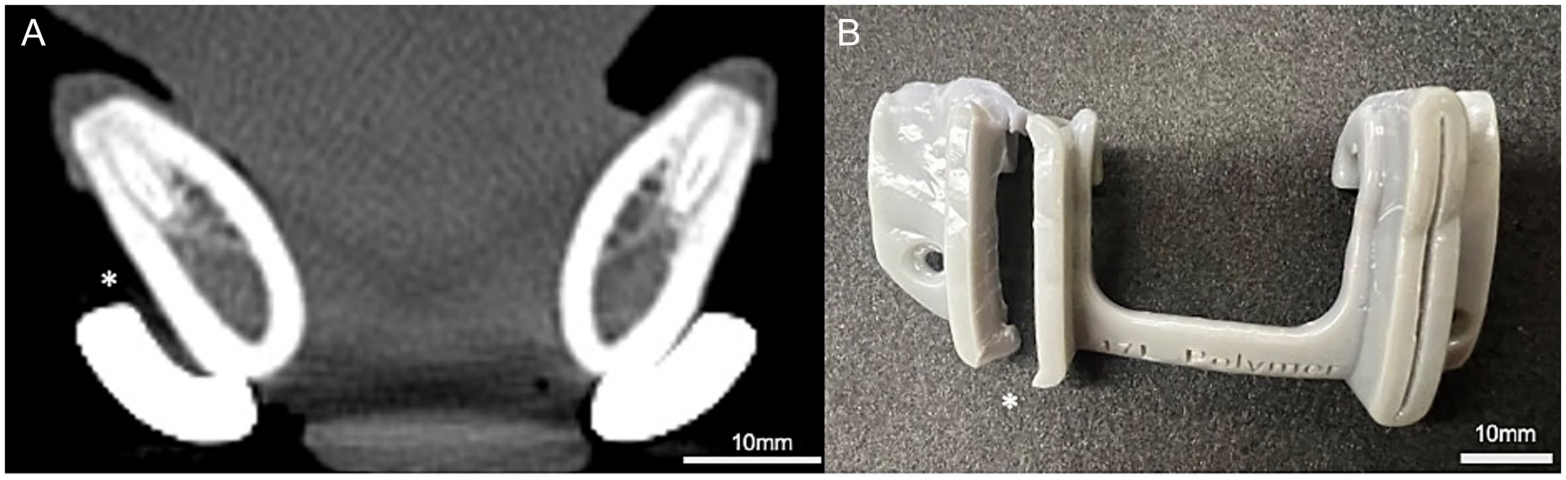
Material-specific limitations. **(A)** Transverse CT view demonstrating a gap (*) between the titanium guide and the mandibular body following placement. **(B)** Picture showing failure by cracking of a polymer guide along the rostral cutting slot following use (*).

Post-placement CT scans revealed dorsal displacement and gap formation of up to 1.9 mm between the guide and mandibular body in 7/20 (35%) of the metal guides. In all cases, the gap was located along the ventral mandibular cortex, with lateral and dorsal deviation of the guide noted at that site (Figure 5). Five of these gaps occurred in procedures performed by the experienced surgeon and two by the novice. In contrast, all polymer guides fit flush to the mandible with no measurable gap. Additionally, post-placement CT scans revealed a median of 3 screw cortices engaged in both metal and polymer groups. The mean attenuation of metal guides was 7039.4 HU, whereas the polymer guides enhanced with 5% barium demonstrated a mean attenuation of 696.5 HU.

### Qualitative assessment

#### Ease of guide placement

Of the 20 metal guides, 12 (60%) were classified as easy to place, 7 (35%) as moderate, and 1 (5%) as difficult. In comparison, 19/20 (95%) polymer guides were rated as easy to place, 1 (5%) as moderate, and none as difficult (Table 1). Polymer guides were associated with significantly easier placement than metal guides (*p* = 0.020). The experienced surgeon rated a higher proportion of guides as easy to place (17/20, 85%) compared to the novice surgeon (14/20, 70%). Placement difficulty was greater for the novice surgeon, though the difference did not reach statistical significance (*p* = 0.450).

**Table 1:** Comparison of ease of guide placement between study groups.

Treatment group	Ease of guide placement		
	Easy	Moderate	Hard
Metal	12/20	7/20	1/20
Polymer	19/20	1/20	0/20
Novice surgeon	14/20	1/20	5/20
Experienced surgeon	17/20	0/20	3/20

Ease of guide placement was compared between material type (metal versus polymer) and surgeon experience (novice versus experienced surgeon). Placement was recorded as *easy* (guide fitted within 30 s without modification of the surgical approach), *moderate* (requiring surgical approach modification and/or fitting within 30 s to 2 min), or *difficult* (unable to fit or stabilize the guide without approach modification and/or fitting requiring more than 2 min).

#### Cut quality

High-quality osteotomies were achieved in 15/20 (75%) of metal-guided cuts and 16/20 (80%) of polymer-guided cuts, with no significant difference between materials (*p* = 1.000). Cuts performed by the experienced surgeon were of significantly higher quality overall compared to the novice (19/20, 95% vs. 12/20, 60%; *p =* 0.020), without influence of material. In the novice group, 8/20 (40%) cuts were graded as moderate quality, while only 1/20 (5%) of cuts in the experienced surgeon group were graded as moderate. No procedures in either group were graded as poor quality.

Incomplete osteotomies were observed in 2/20 (10%) of metal-guided procedures and 1/20 (5%) of polymer-guided procedures. Among the metal guide cases, two procedures had a single incomplete cranioventral cut, and one procedure had two incomplete ventral cuts. No significant differences were found between materials (*p* = 0.605) or between surgeons (*p* = 0.605).

#### Soft tissue trauma and debris

Soft tissue trauma post-osteotomy was mild in 2/40 (5%) procedures and moderate in 3/40 (7.5%). Most procedures (35/40, 87.5%) produced no visible trauma. Post-osteotomy debris was mild in 18/40 (45%) procedures and moderate in 21/40 (52.5%), with no case of severe trauma or debris.

#### Guide failure

No failures occurred with metal guides. In contrast, 9/20 (45%) polymer guides experienced failure (*p* = 0.001) (Figure 5). Of these, 3/9 (33%) were partial failures, characterized by fissuring of the cutting slot, and 6/9 (66%) were complete cracks. Failures occurred in 6/9 (66%) procedures performed by the experienced surgeon and in 3/9 (33%) performed by the novice surgeon, with no significant difference between surgeons (*p* = 0.542). No intraoperative adjustments or alterations in surgical technique were required in cases of partial guide failure, as such failures typically occured near the completion of the osteotomy.

### Efficiency assessment

#### Effect of material

**Figure 6:**
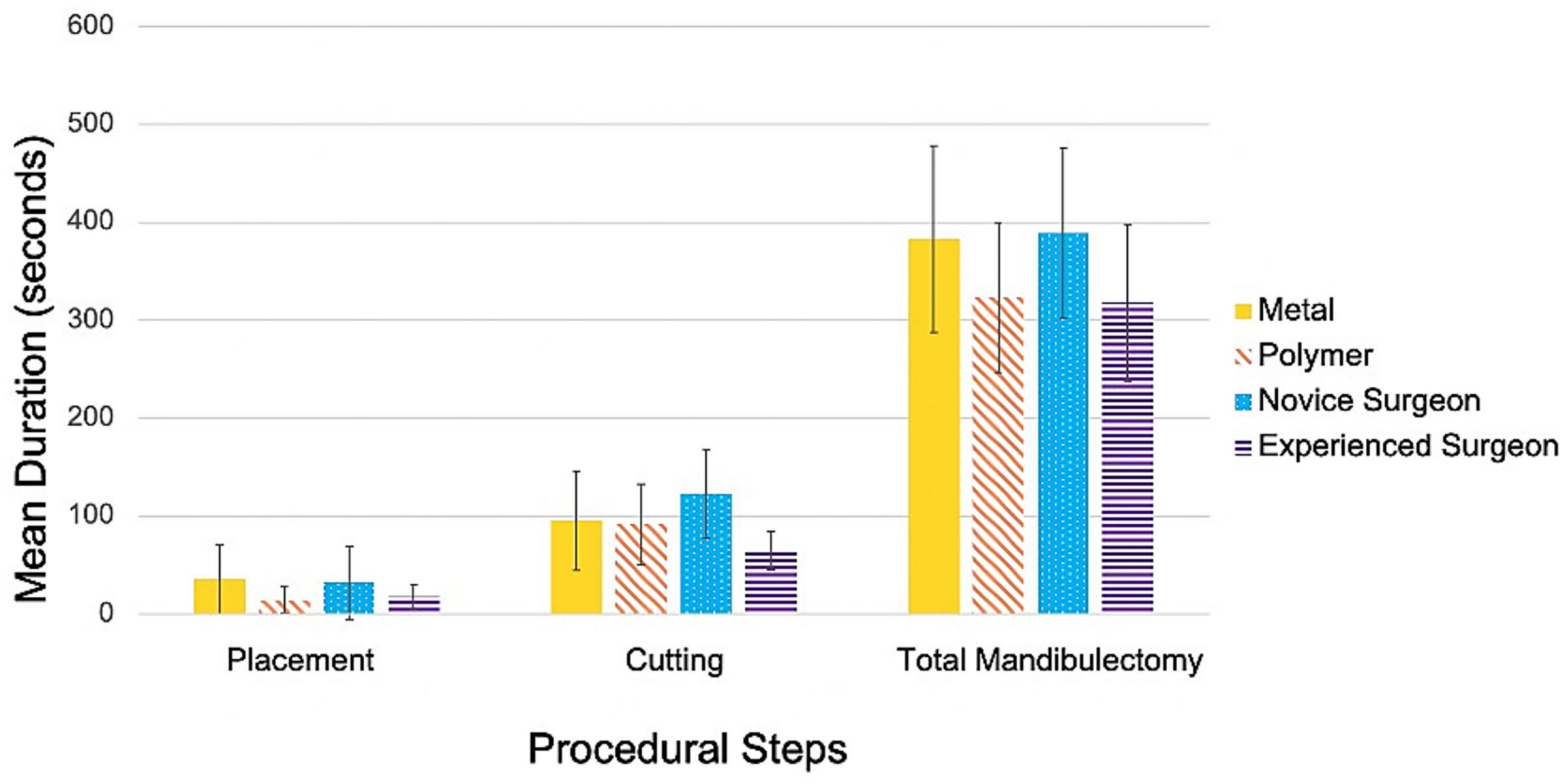
Comparison of procedure times. Mean placement time, cutting time, and total mandibulectomy time compared between material type (metal versus polymer) and surgeon experience (novice versus experienced surgeon).

Significant differences were observed between material types for placement, securing, preparation (placement + securing), and total mandibulectomy times (Figure 6). Mean placement time was significantly longer for metal guides compared to polymer guides (35 s vs. 14 s; *p* = 0.004). Polymer guides were also faster to secure (1.17 × faster; *p* = 0.042), which contributed to a shorter preparation time overall (1.27 × faster; *p* = 0.004). Total mandibulectomy time was 1.19x faster with polymer guides compared to metal (*p* = 0.010).

When stratified by surgeon experience, the difference in total mandibulectomy time between material groups was significant for the experienced surgeon (*p* = 0.006), who required 1.06x longer to perform the procedure with metal guides compared to polymer guides. For the novice surgeon, no significant difference in total procedural time was detected between material types (*p* = 0.970).

#### Effect of surgeon experience

Surgeon experience also significantly influenced procedural times (Figure 6). The experienced surgeon was 1.9x faster than the novice at cutting (*p* = 3.2×10^−6^), 1.43x faster at performing the cut and removing the guide/segment (*p* = 3.0×10^−4^), and 1.23x faster at completing the mandibulectomy (*p* = 0.001). Preparation time (placement + securing) did not differ significantly between surgeons (*p* = 0.180).

The greatest difference in total mandibulectomy time between surgeons was observed with polymer guides (*p* = 0.002), where the experienced surgeon was 1.06x faster than the novice. With metal guides, procedural times did not significantly differ between surgeons (*p* = 0.820).

#### Combined effects of material and surgeon experience

The strongest efficiency difference was between the experienced surgeon using polymer guides and the novice surgeon using metal guides, with the former being 1.07x faster (*p* = 0.001). In contrast, no significant difference in efficiency was observed between the experienced surgeon using metal guides and the novice surgeon using polymer guides (*p* = 0.960).

### Accuracy assessment

The mean absolute linear deviation between the planned and performed cuts was 0.380 ± 0.330 mm. Material type did not significantly influence overall procedural accuracy (*p* = 0.612). A trend was observed toward slightly lower mean cutting error with metal guides (0.382 mm) compared to polymer guides (0.385 mm), though not statistically significant (*p* = 0.817). Positioning error was marginally higher with metal guides (0.473 mm) versus polymer guides (0.434 mm), but again without significance (*p* = 0.677). Similarly, total error was slightly greater for metal guides (0.401 mm) compared to polymer guides (0.368 mm; *p* = 0.612).

**Figure 7:**
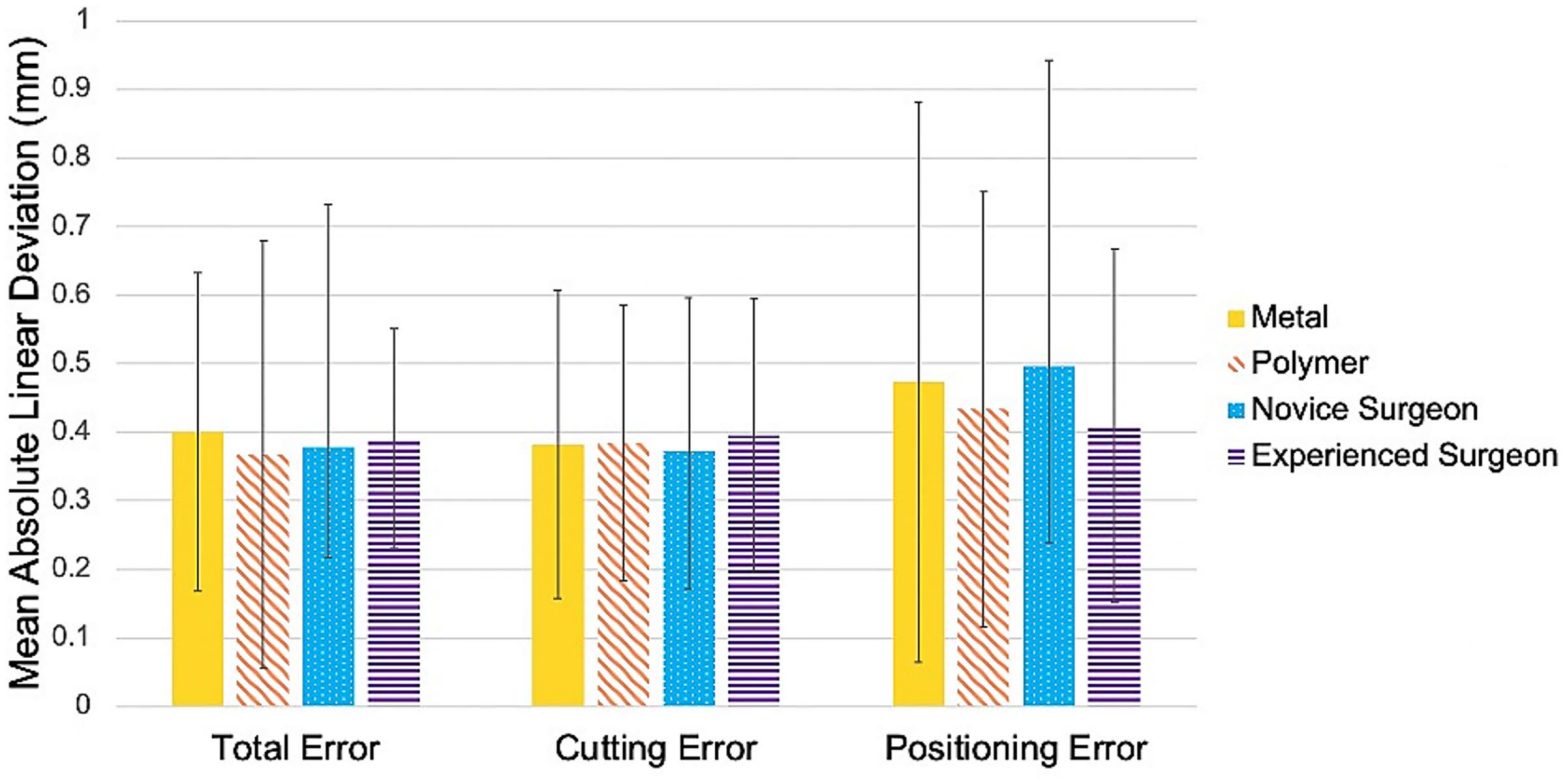
Comparison of average linear deviation. Average positioning, cutting, and total error between guide material (metal versus polymer) and surgeon experience (novice versus experienced). Positioning error refers to the difference between planned and guided osteotomies, cutting error to the difference between guided and performed osteotomies, and total error to the difference between planned and performed osteotomies.

All mean cutting and positioning errors were <0.5 mm, with a mean cutting error of 0.380 ± 0.280 mm and a mean positioning error of 0.450 ± 0.410 mm (Figure 7).

Surgeon experience had no significant effect on overall procedural accuracy between planned and performed cuts (*p* = 0.157), nor on mean cutting error (*p* = 0.660) or positioning error (*p* = 0.790).

However, a significant difference was detected between rostral and caudal cuts, with cutting error in rostral cuts being 1.36 × higher than in caudal cuts (*p* = 0.017). No significant difference in positioning accuracy was observed between the two cut locations (*p* = 0.691).

## Discussion

In this study, we evaluated the influence of material composition (metal versus polymer) on the performance of 3D-printed surgical cut guides designed for segmental mandibulectomy procedures in dog cadavers. Both metal and polymer guides enabled highly accurate osteotomies, with mean linear deviations of <0.5 mm. No significant differences in accuracy were detected between metal and polymer guides, nor between novice and experienced surgeons. Consequently, our hypothesis was rejected as metal guides did not demonstrate superior accuracy for this surgical procedure. However, material-specific limitations were identified: polymer guides exhibited a higher rate of failure due to their flexibility, whereas metal guides posed challenges during intraoperative placement that negatively affected procedural efficiency. These findings highlight potential differences in clinical applicability between the two materials.

Both guides achieved submillimeter accuracy, surpassing previously reported deviations associated with freehand procedures and earlier guide systems in the human literature, where means range from 3.0 to 14.6 mm for freehand pelvic osteotomies (24, 25) and 0.74 to 4.0 mm for guided procedures across various applications (13, 15, 21, 26–28). Similarly, veterinary studies typically report linear deviations of <3 mm for guided osteotomies or implant placement in neurologic, oncologic, and orthopedic applications (3, 7, 29–34). Such precision is particularly advantageous in oncologic applications, where small deviations from the intended margin may result in incomplete excision, higher recurrence risks and poorer prognosis. High accuracy is also critical when osteotomies must align with patient-specific implants to ensure optimal fit.

Consistent with prior studies, surgeon experience did not influence accuracy, supporting the idea that 3D-printed guides help standardize precision across skill level and experience (3, 9, 29, 33). However, experience markedly affected efficiency: the experienced surgeon completed the cutting phase nearly twice as fast as the novice. This efficiency gap was amplified with polymer guides, whereas it diminished with titanium guides, suggesting that material-related handling challenges may mitigate the advantages of surgical experience. Cuts performed by the experienced surgeon were also of higher overall quality, similar to previous studies (3).

A consistent observation was greater error in rostral osteotomies compared to caudal ones. This may reflect increased mobility in the rostral regions relative to the more constrained caudal mandibular guide positioned against the angle of the mandible. Although the present study used a relatively simple mandibulectomy model to minimize sources of error and did not include a freehand comparison, the consistently high accuracy observed suggests that the use of a surgical guide contributes substantially to procedural accuracy. A surgical guide is not strictly required for mandibulectomy procedures performed without reconstruction implants (17); however, this model was selected over a more complex maxillectomy (3) to reduce confounding variables and isolate material composition as the primary factor under investigation.

To our knowledge, this is the first study to directly compare 3D-printed metal and polymer materials for use as surgical cutting guides. Polymer materials, such as acrylate-based resins or nylon-based powders, remain the most commonly used materials, with only a few studies reporting the use of metal guides in human or veterinary surgery (17–19). One previous veterinary study referenced a titanium patient-specific implant intended to function dually as drill guide and implant for lumbosacral stabilization (9, 35). Several studies have also examined the effects of resin formulation, printing technology, and sterilization on custom polymer guide performance (36–38). However, no published study has compared metal and polymer guides and evaluated their relative advantages.

Although accuracy was equivalent between materials, important differences emerged in handling and mechanical performance. Theoretically, metal guides may improve stability due to greater rigidity, reducing micromotion during osteotomy, and improving adherence to the bone through inherent surface roughness without post-processing or polishing. On the other hand, polymer materials may be more suitable when some flexibility is required, such as in press/fit designs, or when transparency is desired to allow visualization. In this study, polymer guides were consistently easier and faster to place, achieved closer conformity to the bone surface and resulted in shorter mandibulectomy times. However, nearly half failed structurally, most commonly by cracking along the cutting slot. Although these failures did not compromise accuracy in this cadaveric model, they remain a concern for clinical use. Metal guides, in contrast, demonstrated excellent mechanical resilience with no observed failures, but their rigidity made placement more difficult and, in some cases, resulted in measurable gaps at the bone–guide interface. Although these gaps did not affect cutting accuracy, they may influence intraoperative stability or surgeon confidence. Design modifications specific to metal guides may include the use of thinner components and walls to improve visibility and manipulation of the construct, with the goal of reducing bulk and enhancing overall fit. This approach would also have the added benefit of decreasing overall material powder usage during manufacturing and reducing total printing costs. Additionally, increased friction, blade dulling, and battery drain appeared to be more inherent to metal guides. These challenges were likely related to the fixed, rigid geometry of the metal guide design. Potential modifications to slot thickness, wall height, blade selection, or the use of a single-wall design may help mitigate these effects and improve equipment longevity. Together, these findings suggest that polymer and metal guides offer distinct trade-offs: polymer guides maximize efficiency and conformity at the cost of durability, whereas metal guides ensure structural integrity but reduced ease of handling.

In all cases in this study, the neurovascular bundle was sharply transected using an oscillating saw. Soft tissue trauma was documented with respect to injury to the gingiva, surrounding musculature, and other adjacent structures, but not specifically to the neurovascular bundle itself. Clinically, hemorrhage would be managed either pre-osteotomy through ligation of the inferior alveolar artery as it enters the mandibular foramen on the medial aspect of the mandible, or post-osteotomy following removal of the surgical guide. Evaluation of piezotome use in conjunction with surgical guides, including its effects on accuracy and direct comparison with an oscillating saw, would be an interesting direction for future investigation, with the potential added benefit of improved control and precision during isolation and ligation of the neurovascular bundle (39, 40). While the human medical literature demonstrates benefits of piezosurgery compared with oscillating saws (41–43), such evidence is currently lacking in the veterinary literature. As a result, ostectomy device choice remains dependent on surgeon preference and institutional practice.

This study has several limitations. The simplified mandibulectomy model and high overall accuracy may have limited the ability to detect subtle differences between groups, raising the possibility of a type II error. A more complex surgical model might reveal greater variability. Likewise, CT slice thickness (0.75 mm) approaches the magnitude of deviations measured (<0.5 mm), introducing inherent quantification limits. Thus, the findings of this study should be interpreted with caution, as the reported differences between groups are small and approach the limits of reliable detection. Guide design was intentionally kept identical across materials to enable direct comparison; however, material-optimized designs could theoretically improve outcomes. For instance, titanium’s strength may allow thinner, intricate guides to enhance conformity in anatomically constrained sites leading to decreased incidence of gap formation, while polymer guides may benefit from reinforced cutting slots to reduce fracture risk. Attempts to modify slot thickness in this study resulted in fusion, and the validated design encompassed the lowest slot thickness that could accommodate a 0.6-mm saw blade. Only one polymer resin and one metal alloy were tested; alternative formulations or processing methods (i.e., annealing, polishing) could yield different results. Screw fixation was chosen to secure the guide, but alternative fixation methods (e.g., pins) may influence stability and efficiency. The effects of sterilization were not investigated as part of the study. Previous studies have investigated the effects of steam sterilization on the dimensional accuracy of three-dimensional printed resin surgical guides, and reported overall dimensional changes related to linear expansion but found no statistically significant impact of steam sterilization on the dimensional accuracy of resin test artifact (38). Further investigation is warranted to assess the effects of steam sterilization on the specific materials and designs used in this study prior to clinical application. Manufacturing times also differed substantially between materials, with polymer guides requiring approximately 4 h per build plate compared to 28 h for metal guides, although these times may vary in clinical scenario depending on plate utilization and material-specific design. Post-processing, particularly for metal guides encountering slot fusion, introduced additional steps that reduced efficiency. Cost remains a major limitation to clinical accessibility, with metal printers costing 30 to 200 times more than polymer printers,^1^^,^^2^ limiting accessibility outside of high-volume production or academic centers. Finally, the cadaveric design limits generalizability to clinical practice. Live surgical environment introduces additional challenges, including hemorrhage, thermal injury, soft tissue retraction, margin determination or space-occupying lesion not represented in this model.

Both polymer and metal surgical guides achieved submillimeter accuracy independent of surgeon experience, reinforcing the potential of additive manufacturing to standardize surgical outcomes and support training. However, given the lack of accuracy advantage with titanium, the costs, technical demands, and production time of metal 3D-printing may not be justified for straightforward procedures. Material choice should therefore be application specific. Polymer guides appear well suited for procedures requiring rapid placement, ease of handling, and close conformity, provided material durability can be improved. On the other hand, metal guides may be advantageous in high-stress applications such as orthopedic surgery, where mechanical resilience is critical, but may require refinements to improve conformity and handling. Integrating material-specific design considerations will be key to optimizing guide performance across diverse surgical contexts. Future research should explore alternative materials, high-strength polymers or composites, varied additive manufacturing technologies, and performance in live surgical settings involving reconstruction, margin assessment, and long-term outcomes.

In conclusion, both metal and polymer 3D-printed surgical guides achieved submillimeter accuracy in mandibulectomy procedures in the dog, with mean linear deviations <0.5 mm regardless of material or surgeon experience. Material choice did not influence accuracy but did affect usability, durability, and efficiency. Titanium metal guides were significantly more expensive and time consuming to manufacture compared to polymer guides. These findings underscore the importance of tailoring guide design to material characteristics and clinical priorities rather than assuming inherent superiority of one material over another.

## Data Availability

The raw data supporting the conclusions of this article will be made available by the authors, without undue reservation.

## Footnotes

1
https://www.3dsystems.com/how-to-buy

2
https://formlabs.com/store/3d-printers/form-4/?srsltid=AfmBOorGvgjpEIktpwrWazPp0ircBREcNPsT3YAANiokn9cmOxrjehFC#/
